# Association Between Glaucoma and Brain Structural Connectivity Based on Diffusion Tensor Tractography: A Bidirectional Mendelian Randomization Study

**DOI:** 10.3390/brainsci14101030

**Published:** 2024-10-17

**Authors:** Lian Shu, Xiaoxiao Chen, Xinghuai Sun

**Affiliations:** 1 Department of Ophthalmology & Visual Science, Eye & ENT Hospital, Shanghai Medical College, Fudan University, Shanghai 200031, China; lshu14@fudan.edu.cn (L.S.); xxchen_clara@hotmail.com (X.C.); 2 State Key Laboratory of Medical Neurobiology and MOE Frontiers Center for Brain Science, Institutes of Brain Science, Fudan University, Shanghai 200032, China; 3 NHC Key Laboratory of Myopia, Chinese Academy of Medical Sciences, and Shanghai Key Laboratory of Visual Impairment and Restoration (Fudan University), Shanghai 200031, China

**Keywords:** glaucoma, diffusion tensor tractography, diffusion tensor imaging, Mendelian randomization, structural connectivity, neurodegenerative disease

## Abstract

Background: Glaucoma is a neurodegenerative ocular disease that is accompanied by cerebral damage extending beyond the visual system. Recent studies based on diffusion tensor tractography have suggested an association between glaucoma and brain structural connectivity but have not clarified causality. Methods: To explore the causal associations between glaucoma and brain structural connectivity, a bidirectional Mendelian randomization (MR) study was conducted involving glaucoma and 206 diffusion tensor tractography traits. Highly associated genetic variations were applied as instrumental variables and statistical data were sourced from the database of FinnGen and UK Biobank. The inverse-variance weighted method was applied to assess causal relationships. Additional sensitivity analyses were also performed. Results: Glaucoma was potentially causally associated with alterations in three brain structural connectivities (from the SN to the thalamus, from the DAN to the putamen, and within the LN network) in the forward MR analysis, whereas the inverse MR results identified thirteen brain structural connectivity traits with a potential causal relationship to the risk of glaucoma. Both forward and reverse MR analyses satisfied the sensitivity test with no significant horizontal pleiotropy or heterogeneity. Conclusions: This study offered suggestive evidence for the potential causality between the risk of glaucoma and brain structural connectivity. Our findings also provided novel insights into the neurodegenerative mechanism of glaucoma.

## 1. Introduction

As a neurodegenerative ocular disease, glaucoma is characterized by the progressive death of retinal ganglion cells (RGCs) and structural changes in the optic disc, resulting in visual field defects. It is the primary cause of irreversible blindness globally [1]. Although elevated intraocular pressure (IOP) is the main known risk factor of glaucoma, the onset or progression of glaucomatous damage can still occur in a normal IOP state [2]. Thus, novel biomarkers and therapeutic strategies of glaucoma are urgently needed at present. However, the pathogenesis and mechanism of glaucomatous neurodegeneration are complex and not fully understood. Recent neuroimaging and neuropathological evidence from both animal models and patients have suggested that the damage of glaucoma extends beyond the visual system of the brain [3,4,5], and glaucoma is being considered as a neurodegenerative brain disorder [6,7].

Brain tissue can be divided into white matter (WM) and gray matter (GM), with the former composed of neurons and the latter composed of bundles of myelinated nerve fibers. Exploring the structures of WM contributes to our better understanding of the complex structural connectomes among different brain neurons [8]. Due to the fact that water molecules prefer to diffuse along axonal bundles rather than across them, diffusion tensor imaging (DTI) can value the integrity of WM from microstructural aspects by detecting various diffusion properties based on diffusion magnetic resonance imaging (dMRI) [9,10]. Furthermore, through three-dimensional reconstruction of DTI data, diffusion tensor tractography (DTT) reveals the spatial and structural location of WM fibers and evaluates brain structural connectivity effectively and noninvasively [11,12], providing novel insights into the mechanism of central nerve system (CNS) diseases.

DTI and DTT studies of glaucoma have been widely conducted in recent years. Some DTI-based neuroimaging studies have confirmed the presence of structural brain neurological alterations in patients with glaucoma, especially in the visual pathway [13,14,15]. For example, the fractional anisotropy of DTI was significantly lower, while the mean and radial diffusivity were significantly higher in the optic nerve and radiations in patients with glaucoma [14,16,17]. Additionally, widespread disruption of the brain structural connectome was reported in recent DTT studies on glaucoma, involving complex cognitive and behavioral dysfunctions [18,19,20]. Despite the strong correlation between glaucoma and abnormalities in brain structural connectivity, our understanding of the extra causal association remains limited.

Advances in genome-wide association studies (GAWSs) and the deep exploration of the single nucleotide polymorphisms (SNPs) benefit the development of Mendelian randomization (MR) analysis. This method applies genetic variations as natural instruments and is widely employed in studying causal relationships between different diseases or phenotypes [21,22]. The reliability of evidence for causal relationships based on MR studies is normally considered to be intermediate between observational and experimental epidemiologic studies. Recently, MR analyses between glaucoma and different diseases or phenotypes have also been reported widely to identify potential risk factors [23,24]. However, brain structural connectivity has not yet been included.

Therefore, we conducted a bidirectional MR study to explore the causal relationships between glaucoma and brain structural connectivity [25]. This study aims to offer novel insights into the glaucomatous neurodegenerative mechanisms, and identify potential therapeutic strategies for glaucoma.

## 2. Materials and Methods

### 2.1. GWAS Data and Design of This Study

The characteristics of GWAS data of glaucoma and DTT are shown in Table 1. The definition of glaucoma in this study was based on the code category H40 in the International Classification of Diseases-10. Publicly available GWAS data were sourced from the FinnGen database (R10) for glaucoma, with 20,906 cases and 391,275 controls included. The GWAS data of DTT were recently reported [25] and accessed from the GWAS catalog. A total of 206 brain structural connectome phenotypes derived from DTI tractography in 26,333 UK Biobank participants were obtained. These imaging-derived phenotypes represented the density of myelinated interconnections among three types of measures: (1) seven connectomics-based networks (visual network [VN], default mode network [DMN], salience/ventral attention network [SAN/VAN], dorsal attention network [DAN], control network [CN], limbic network [LN], and somatomotor network [SN]); (2) left hemisphere (LH) and right hemisphere (RH); and (3) seven subcortical regions: accumbens, amygdala, caudate, thalamus, hippocampus, pallidum, and putamen [26]. Details of the 206 DTT phenotypes are showed in Supplementary Table S1.

**Figure 1:**
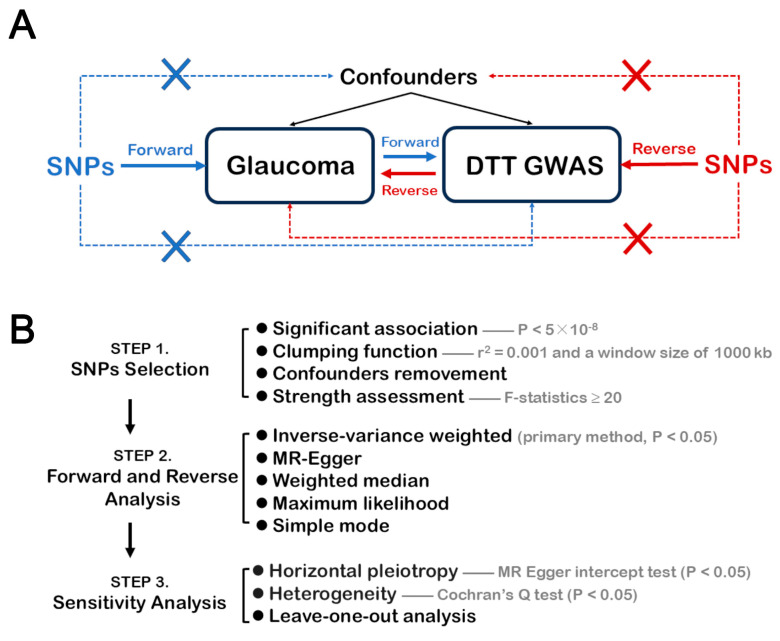
Study design and process of MR analysis. (**A**) Study design of bidirectional MR analysis between glaucoma and 206 traits based on DTT GWASs; (**B**) Process of MR analysis in three brief steps: SNPs selection, forward and reverse MR analysis, and sensitivity analysis. SNPs, single nucleotide polymorphisms; MR, Mendelian randomization; DTT, diffusion tensor tractography; GWAS, genome-wide association studies.

MR analysis depends on three core principles: (1) genetic variations must have a significant association with the exposure factor; (2) genetic variation must impact the outcome only through the exposure factor; and (3) genetic variations must be independent of all confounding factors. The basic study design and the general MR analysis process is shown in Figure 1. We conducted a bidirectional MR analysis between glaucoma and 206 traits based on DTT GWASs to explore the causal relationships between glaucoma and brain structural connectivity. In forward MR analysis, glaucoma risk was used as the exposure factor and alterations in brain structural connectivity were used as the results. Conversely, brain structural connectivity was used as the exposure factor, while glaucoma risk was used as the outcome in reverse MR analysis. In order to enhance the interpretation and convince others of our MR results, we strictly followed the STROBE-MR (https://www.strobe-mr.org/, accessed on 29 August 2024, a guideline that contains the necessary processes and details for reporting MR studies [27].

### 2.2. Selection of SNPs

Based on the assumptions of MR analysis, the following procedures of our SNP selection were employed for instrumental variables (IVs): (1) associated with exposure with significance as *p* < 5 × 10^−8^; (2) using a clumping function with the following parameters of linkage-disequilibrium pruning: r^2^ = 0.001 with the window size of 1000 kb to screen for independent SNPs [28]; (3) removing the SNPs associated with confounders that affect the outcomes (such as intraocular pressure, obesity, myopia) [24] based on the PhenoScanner v.2 databases; and (4) assessing the strength of SNPs as instrumental variations by calculating the F-statistics and ensuring F-values ≥ 20.

### 2.3. Statistical Analysis

We applied the random effect-based inverse-variance weighted (IVW) method as the primary tool for assessing the association between glaucoma and brain structural connectivity [29], and an IVW *p*-value < 0.05 was regarded as suggestive of potential causality. The false discovery rate (FDR) was used to adjust the results as the q-value, and the results were considered as significant when the FDR < 0.05. Additional statistical analyses, including the maximum likelihood test, simple mode, weighted median, and MR–Egger, were conducted to obtain robust estimates of causality. The odds ratio (OR) indicated the effect degree of the causal associations between exposure and outcome.

Further sensitivity analysis and assessment were performed on the forward and reverse MR analyses. The control and avoidance of horizontal pleiotropy is one of the key assumptions in MR studies. The intercept in MR–Egger’s regression can be interpreted as an estimate of the average pleiotropy of the entire genetic variance [30]. Thus, horizontal pleiotropy of SNPs was detected via the MR–Egger intercept test (*p* < 0.05). Cochran’s Q test was applied to detect the degree of variation among SNPs and avoid heterogeneity (*p* < 0.05) [31]. Additionally, to ensure the stability and reliability of our MR results, leave-one-out analysis was utilized to remove and re-estimate each SNP [28]. Specifically, the results were considered robust when the error bars were all on the same side of the zero line in the leave-one-out analysis. All analyses in our study were conducted using R software (version 4.3.2) and the “TwoSampleMR packages” (28).

## 3. Results

### 3.1. Study Overview

Through forward and reverse MR analyses, we identified three and thirteen DTT traits (IVW: *p* < 0.05) potentially serving as outcomes and risk factors of glaucoma, respectively. These findings offer suggestive evidence for the potential causal relationships between glaucoma and brain structural connectivity. All results of the bidirectional MR analysis are presented in Supplementary Tables S2 and S3, respectively.

### 3.2. Forward MR Analysis

**Figure 2:**
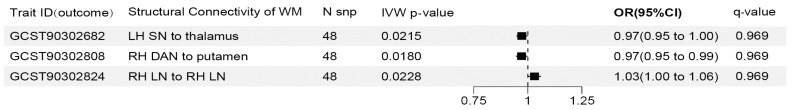
Causalities in the forward MR analysis between glaucoma and DTT traits. An IVW *p*-value < 0.05 was regarded as suggestive of causality and the false discovery rate (FDR) was used to adjust the results as the q-value. SNP, single nucleotide polymorphism; WM, white matter; RH, right hemisphere; LH, left hemisphere; IVW, inverse-variance weighted; CI, confidence interval; OR, odds ratio; SN, salience network; DAN, default mode network; LN, limbic network.

The results of our forward MR analysis suggested that glaucoma could lead to brain structural connectivity abnormalities, mainly in three DTT traits: GCST90302682, GCST90302808, and GCST90302824 (Figure 2). Our study indicated that glaucoma was negatively associated with brain structural connectivity from the LH SN to the thalamus (IVW OR = 0.972, 95% confidence interval [CI]: 0.949–0.996, *p* = 0.0215) and from the RH DAN to the putamen (IVW OR = 0.971, 95% CI: 0.947–0.995, *p* = 0.0180), whereas it was positively related to changes in brain structural connectivity from the RH LN to the RH LN (IVW OR = 1.031, 95% CI: 1.004–1.058, *p* = 0.0228). Additional analyses by the maximum likelihood test, simple mode, weighted median, and MR–Egger also demonstrated consistent causal estimates in the same direction (Supplementary Table S2). However, no significant causality was observed after the FDR adjustment (q > 0.05) for any phenotype. These three DTT traits offered novel evidence for the potential forward causality between glaucoma and DTT phenotypes, illustrating the impact of glaucoma on brain structural connectivity.

### 3.3. Reverse MR Analysis

**Figure 3:**
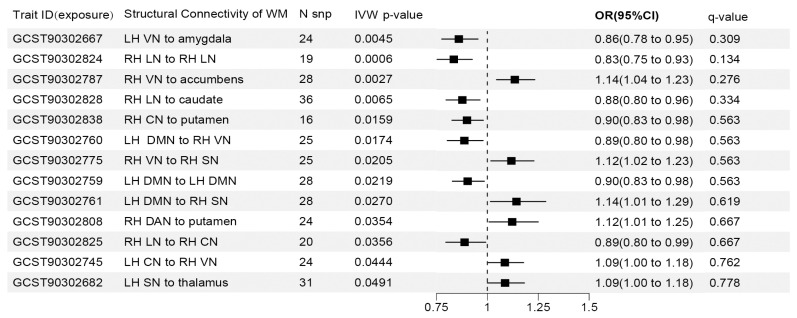
Causalities in the reverse MR analysis between glaucoma and DTT traits. An IVW *p*-value < 0.05 was regarded as suggestive of causality and the false discovery rate (FDR) was used to adjust the results as the q-value. SNP, single nucleotide polymorphism; WM, white matter; RH, right hemisphere; LH, left hemisphere; IVW, inverse-variance weighted; CI, confidence interval; OR, odds ratio; DMN, default mode network; SN, salience network; DAN, default mode network; VN, visual network; LN, limbic network; CN, control network.

Our reverse MR study identified 13 DTT phenotypes potentially causally related to the risk of glaucoma. Details of each brain structural connectivity are shown in Figure 3. The three most significant traits were GCST90302667, GCST90302824, and GCST90302787, representing connectivity from the LH VN to the amygdala (IVW OR = 0.860, 95% CI: 0.776–0.954, *p* = 0.0045), from the RH LN to the RH LN (IVW OR = 0.835, 95% CI: 0.753–0.926, *p* = 0.0006), and from the RH VN to the accumbens (IVW OR = 1.135, 95% CI: 1.045–1.233, *p* = 0.0027), respectively. An additional four analyses indicated that the causal estimates were in a consistent direction with the IVW method (Supplementary Table S3). However, after FDR correction, we obtained the same insignificant results as those in the forward MR analysis (q > 0.05). Therefore, these findings suggested the potential causality between DTT phenotypes and glaucoma, revealing the influence of brain structural connectivity on glaucoma risk.

### 3.4. Sensitivity Analysis

**Figure 4:**
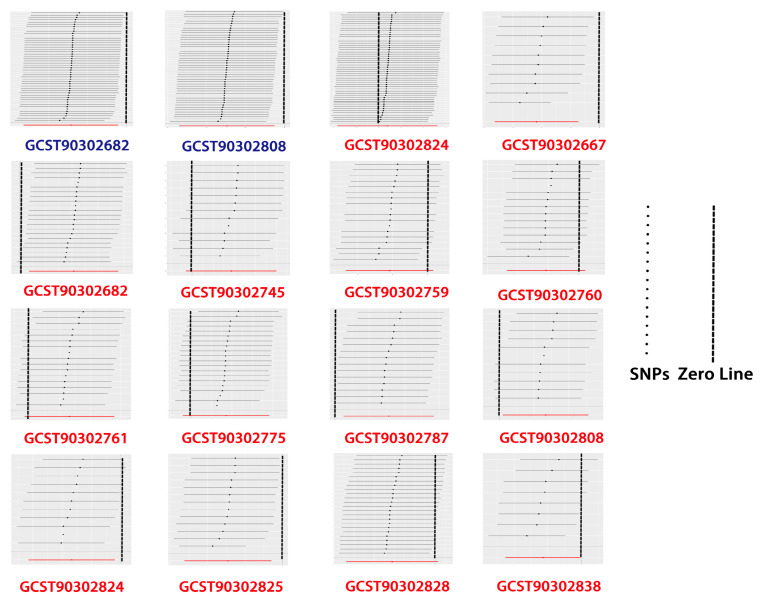
Leave-one-out analysis of the forward (blue) and reverse (red) MR results. The results were considered robust when the error bars of SNPs were all on the same side of the zero line in leave-one-out analysis.

No significant heterogeneity or heterogeneity was revealed in either forward or reverse MR analysis according to our MR–Egger intercept test (*p* > 0.05) and Cochran’s Q test (*p* > 0.05), as shown in Table 2. Furthermore, none of the traits identified from the forward or reverse MR analyses showed significant SNPs driving the causality between glaucoma and brain structural connectivity according to our leave-one-out analysis (Figure 4, Supplementary File S1).

## 4. Discussion

In contrast to traditional anatomical MRI, advanced brain imaging techniques based on dMRI, such as DTI and DTT, can help us understand the structural changes in brain degenerative diseases more effectively and thoroughly. Although DTI can reveal abnormalities in the microstructure of WM, it cannot reflect the structural connectivity features responsible for signal transmission between neurons. DTT further compensates for the shortcomings of DTI and plays an essential role in mapping and constructing the comprehensive “connectomics” [18,32]. Growing evidence from DTT studies has suggested that glaucoma involves the disruption of white matter structural connectivity or networks beyond the visual system [20,33,34]. However, these cross-sectional studies have inherent limitations, including small sample sizes, high patient heterogeneity, and poor reproducibility. To our knowledge, this is the first study to investigate the association between glaucoma and brain structural connectivity based on DTT using MR analysis.

The forward MR analysis results suggested that glaucoma could induce abnormalities in brain structural connectivity in three pairs of regions: from the SN to the thalamus, from the DAN to the putamen, and within the LN network. Previous functional MRI studies have revealed common alterations in brain functional connectivities in the SN, DAN, and LN among patients with glaucoma [6,35,36]. These findings reflect the combined impact of structural and functional WM changes [37]. Volume reductions in the thalamus and putamen were also found in patients with glaucoma based on traditional structural MRI [38]. Notably, both clinical studies and animal model research have shown that structural and functional damage to the thalamus plays an essential role in glaucomatous nerve damage [39,40,41] because the thalamus is a higher sensory center and the intermediate nucleus of visual signals containing the RGC projection region, the dorsal lateral geniculate nucleus (dLGN) [42]. These changes in structural connectivity may result from forward trans-synaptic neurodegeneration during glaucomatous damage [43]. Damage to the RGCs, the core pathological injury in glaucoma, can extend along the visual pathway, resulting in abnormal structural connectivity in the CNS. This may involve neuronal glial activation or demyelination, as suggested by previous studies on neurodegenerative optic radiation before and after dLGN [44,45]. Additionally, the loss of visual signals in patients with glaucoma may result in abnormalities of the brain connectivities beyond the visual pathway [46]. However, it is worth noting that the effect size of the causal associations between exposure and outcome were relatively small in our forward analysis. It could be caused by the winner’s curse [47,48], an overestimation of the effects between SNPs and exposure. Considering the prior observational studies on neuroimaging in patients with glaucoma and animal models [46,49], our forward MR results confirmed the prior findings and provided stronger evidence for the potential forward causal relationship between glaucoma and brain structural connectivity.

Our reverse MR analysis results showed 13 structural connectivity abnormalities between the visual network and nonvisual networks/regions, as well as between nonvisual networks/regions themselves, all of which could potentially influence the risk of glaucoma. These findings indicate that glaucoma belongs to a neurodegenerative brain disorder that may originate from the CNS. In a dMRI-based MR study, Liu et al. [50] suggested that altered microstructural properties of WM might be involved in glaucoma, and our results further confirmed these prior findings. Among the three most significant DTT traits, a 1 standard deviation (SD) increase in the enhanced density of structural connectivity from the LH VN to the amygdala, as well as from the RH LN to the RH LN, reduced the risk of glaucoma by 14% and 16.5%, respectively. Conversely, a 1 SD increase in the density of structural connectivity from the RH VN to the accumbens increased the risk of glaucoma by 13.5%. Among these networks and regions, the amygdala and accumbens, connected to the VN, play essential roles in emotional regulation [51,52]. Recently, Chen et al. [53] found extensive abnormalities in the structural connectivity between the amygdala and other brain regions, such as the cerebellum, middle frontal gyrus, and putamen, in patients with glaucoma. The authors suggested that these might be related to psychomotor dysfunction. Reverse trans-synaptic degeneration may involve VN-related brain structural connectivity abnormalities [43]; however, the mechanisms by which non-VN-related connectivity abnormalities affect glaucoma remain unknown. Researchers have proposed the network degeneration hypothesis to explain the onset of some neurodegenerative brain diseases, which is that they may progress along neuronal brain networks [54,55]. Our findings provided a novel direction and target for further investigations of the specific pathological mechanisms of this hypothesis.

No significant differences were observed in either forward or reverse MR analyses after FDR correction. This was relatively common in previous large-sample MR studies and might be related to our stringent filtering criteria and analytical methods [56,57]. Our sensitivity analyses (Cochran’s Q test, the MR–Egger intercept test, and the leave-one-out test) confirmed the stability and reliability of our bidirectional MR results. Thus, we concluded that this study offered important evidence for the potential causality between glaucoma and brain connectivity, while more rigorous clinical or experimental studies are still needed to confirm that.

This study confirmed that visual pathway damage and abnormal brain structural connectivity are an essential part of glaucomatous neurodegeneration. It deepens our understanding of the glaucomatous neurodegenerative mechanism. Therefore, simply reducing intraocular pressure (IOP) may not be the only reliable approach for preventing and treating glaucoma, and it also explains the occurrence and progression of glaucoma within normal IOP clinically. Limited clinical and animal evidences have suggested that improving visual function may induce neuroregeneration of white and gray matter in the visual pathway [49,58,59]. Thus, our findings may contribute to guiding therapeutic targets that expand from the retina to the CNS, and stimulating the development of novel therapeutic strategies based on neuroprotection, neuroplasticity, and regenerative medicine [49,60,61,62]. This study also suggested the wide prospects of dMRI or DTT in the clinical application for glaucoma. As a potential imaging biomarker, DTT may be suitable for the early and differential diagnosis of glaucoma [9,20,63,64]. Although previous studies indicated that changes in DTI are associated with the severity of glaucoma [65,66], utilizing DTI to assess the severity of glaucoma is economically inefficient, as we have more efficient ophthalmic examinations such as visual field tests. Notably, DTI measurements are actually not specific to myelin or axonal injuries in white matter and lack a more precise biological interpretation [13]. Therefore, the anatomical or pathological processes underlying the microstructural changes in the brain that potentially occur as an outcome or risk factor of glaucoma remain incompletely understood. Furthermore, the absence of standardized imaging analysis protocols (sequences or algorithms) may also restrict the clinical application of DTI in glaucoma. Therefore, improving the dMRI techniques and processing algorithms for DTT data will also be the focus of future studies. Indeed, multimodal MRI studies (including traditional structural MRI, dMRI, and functional MRI) have revealed widespread structural, functional, and metabolic brain connectivity alterations in patients with glaucoma [67,68]. We believe that more imaging biomarkers for the diagnosis or treatment of glaucoma would be found in the future, with advancements in DTT and its integration with other MRI methods.

In fact, altered structural brain connectivity is also observed in other ocular diseases, such as myopia, macular degeneration, and amblyopia [69,70,71]. Although a specific causal relationship needs to be identified, these findings suggested the existence of a brain–eye axis [50,72]. The retina is the outward extension of the CNS, and the eye and the brain share a similar anatomy, histology, and physiology. Ocular diseases may be associated with neurodegenerative diseases of the CNS [73,74,75]. Conversely, neurodegenerative brain disorders, such as Alzheimer’s disease, are commonly accompanied by retinal structure disorders [76,77,78]. Therefore, the strong correlation between eye and brain diseases has long been a popular topic in neurological research [72]. The glaucoma-related brain structural connectivities in our study could be potential targets for further exploration of the brain–eye axis, improving both our understanding and management of brain–eye integration disorders.

Although Mendelian randomization is an advancing robust genetic epidemiology tool to explore causal associations, it has many inherent pitfalls and complications [79]. Firstly, although our study employed certain analytical methods to detect and avoid pleiotropy, the effectiveness remains limited; second, the onset and progression of glaucoma is typically a dynamic process, and our MR analysis is based on cross-sectional studies, making it difficult to capture the temporal dynamic associations between glaucoma and brain structural connectivity; third, confounding factors or biases arising from complex gene–environment interactions are difficult to control, especially when applying GWASs to studies on social behavior or psychological activities [80]. Considering the widespread presence of emotional issues in patients with glaucoma [81,82], we need to be very cautious when making causal inferences between glaucoma and brain structural connectivity through MR studies, where psychiatric disorders can directly lead to brain structural and functional remodeling [83]; and fourth, issues of data sources and population distribution in MR studies also require attention. The GWAS data were not collected with our study objectives in mind, but rather sourced from the potentially biased and unstable-quality databases, which may compromise our statistical power. Additionally, it limits our interpretation of the original data and further subgroup analyses for glaucoma, because acute and chronic IOP elevation might affect different visual pathways in patients with glaucoma [84,85]. Moreover, our study may have restricted generalizability due to its focus on a specific European population. Thus, more large-scale clinical studies based on diverse populations are needed. However, this presents a double-edged sword in balancing generalizability against more confounding factors.

Last but not the least, conclusive causality between glaucoma and brain structural connectivity cannot be established by our MR study alone. Actually, the results of this MR study should be interpreted in the light of evidence from other studies, such as traditional observational clinical trials and experimental research in human and animal models. In a word, conclusive causal inference requires triangulation of evidence [79]. Importantly, MR analysis can provide supportive evidence for causal inference, especially when a randomized controlled trial (RCT) cannot be conducted, and that is why we chose MR analysis to investigate the potential associations between glaucoma and brain structural connectivity.

## 5. Conclusions

Overall, our study identified different DTT phenotypes, which provided suggestive evidence for the potential causal associations between glaucoma and brain structural connectivity. Additionally, this study provided novel insights into glaucomatous neurodegenerative mechanisms, and indicated potential neuroimaging biomarkers and neurotherapeutic strategies for glaucoma.

## Figures and Tables

**Table 1:** Characteristics of GWAS data of glaucoma and 206 DTT traits.

Traits	Year	Database	Data Sources	Sample Sizes	Population
Glaucoma	2021	FinnGen.R10	https://storage.googleapis.com/finngen-public-data-r10/summary_stats/finngen_R10_H7_GLAUCOMA.gz (accessed on 29 August 2024);	20,906 cases and 391,275 controls	EUR
DTT traits	2024	UK Biobank	accession numbers GCST90302648 to GCST90302853; https://www.ebi.ac.uk/gwas (accessed on 29 August 2024);	26,333 individuals	EUR

GWASs, genome-wide association studies; DTT, diffusion tensor tractography; EUR, European.

**Table 2:** Our MR–Egger intercept and Cochran’s Q test of our bidirectional MR analysis.

Traits ID	Structural Connectivity	N(SNPs)	Egger Intercept	*p*-Value of Egger Intercept	Cochran’s Q	*p*-Value of Cochran’s Q
Forward MR						
GCST90302682	LH SN to thalamus	48	−0.002973	0.536	31.50	0.960
GCST90302808	RH DAN to putamen	48	0.002980	0.540	29.28	0.980
GCST90302824	RH LN to RH LN	48	0.000211	0.966	34.11	0.920
Reverse MR						
GCST90302667	LH VN to amygdala	24	0.003648	0.677	16.09	0.851
GCST90302824	RH LN to RH LN	19	0.010224	0.414	10.54	0.913
GCST90302787	RH VN to accumbens	28	0.001613	0.830	17.22	0.926
GCST90302828	RH LN to caudate	36	0.012419	0.086	32.93	0.568
GCST90302838	RH CN to putamen	16	0.000023	0.998	6.68	0.966
GCST90302760	LH DMN to RH VN	25	−0.009642	0.197	19.19	0.742
GCST90302775	RH VN to RH SN	25	0.010420	0.203	19.47	0.727
GCST90302759	LH DMN to LH DMN	28	0.005086	0.425	17.46	0.919
GCST90302761	LH DMN to RH SN	28	0.008127	0.296	25.46	0.549
GCST90302808	RH DAN to putamen	24	−0.010452	0.141	23.74	0.419
GCST90302825	RH LN to RH CN	20	0.004533	0.569	13.98	0.785
GCST90302745	LH CN to RH VN	24	−0.002204	0.756	13.43	0.942
GCST90302682	LH SN to thalamus	31	0.004978	0.504	19.85	0.920

MR, Mendelian randomization; SNPs, single nucleotide polymorphisms; LH, left hemisphere; RH, right hemisphere; SN, salience network; DAN, default mode network; LN, limbic network; VN, visual network; DMN, default mode network; CN, control network. *p* < 0.05 was regarded as significant.

## Data Availability

All statistical GWAS and MR data are publicly available in this study and provided in this paper or the Supplementary Material.

## Supplementary Materials

The following supporting information can be downloaded at https://www.mdpi.com/article/10.3390/brainsci14101030/s1. Table S1: Details of the 206 DTT phenotypes; Table S2: All results of the forward MR analyses; Table S3: All results of the reverse MR analyses; File S1: Detailed results of the leave-one-out analysis.
