# Supplementary figures and images for: Association Between Glaucoma and Brain Structural Connectivity Based on Diffusion Tensor Tractography: A Bidirectional Mendelian Randomization Study
Source: Brain Sci. 2024 Oct 17;14(10):1030. doi: 10.3390/brainsci14101030 (PMC11506416; doi:10.3390/brainsci14101030)

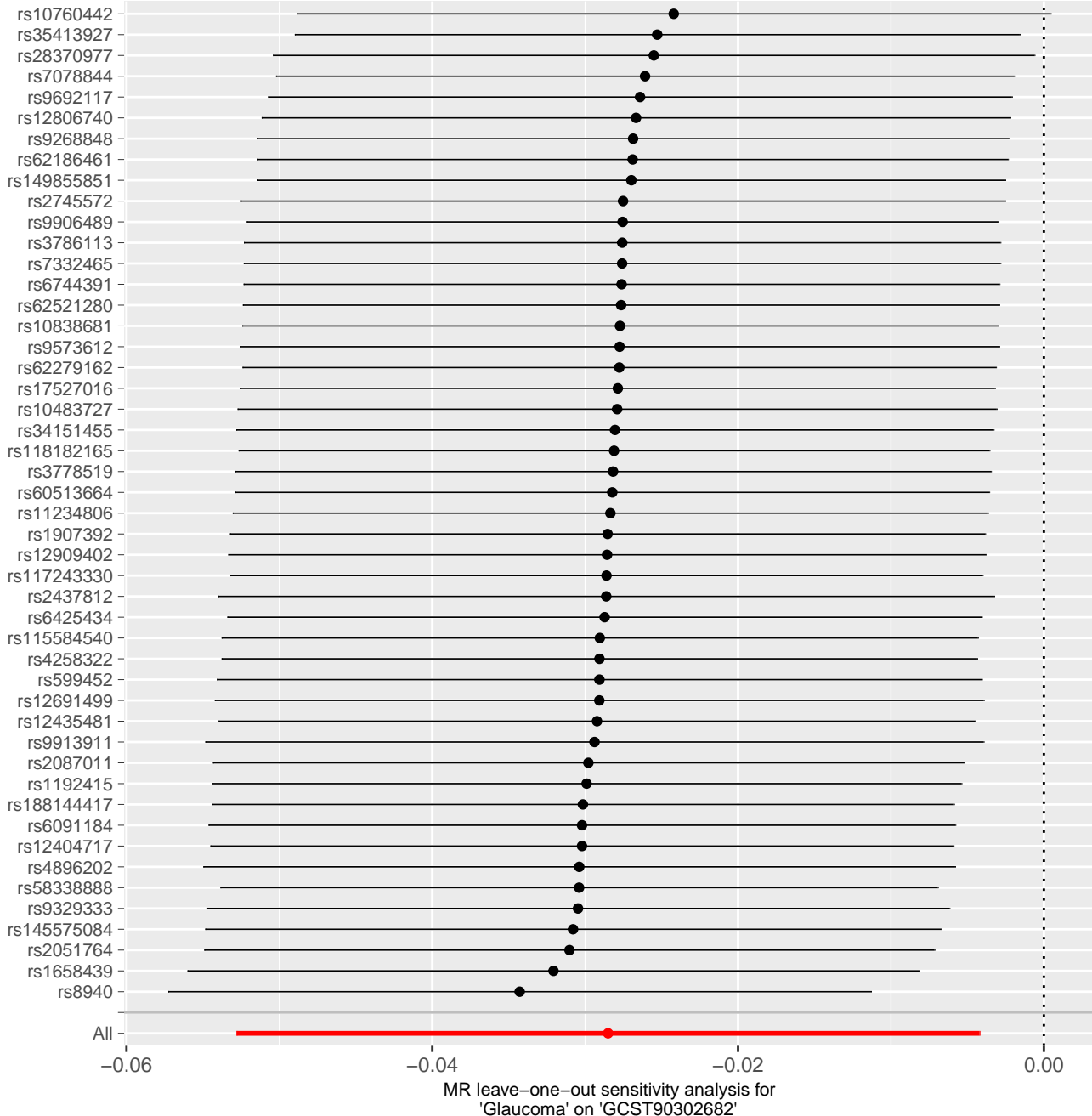

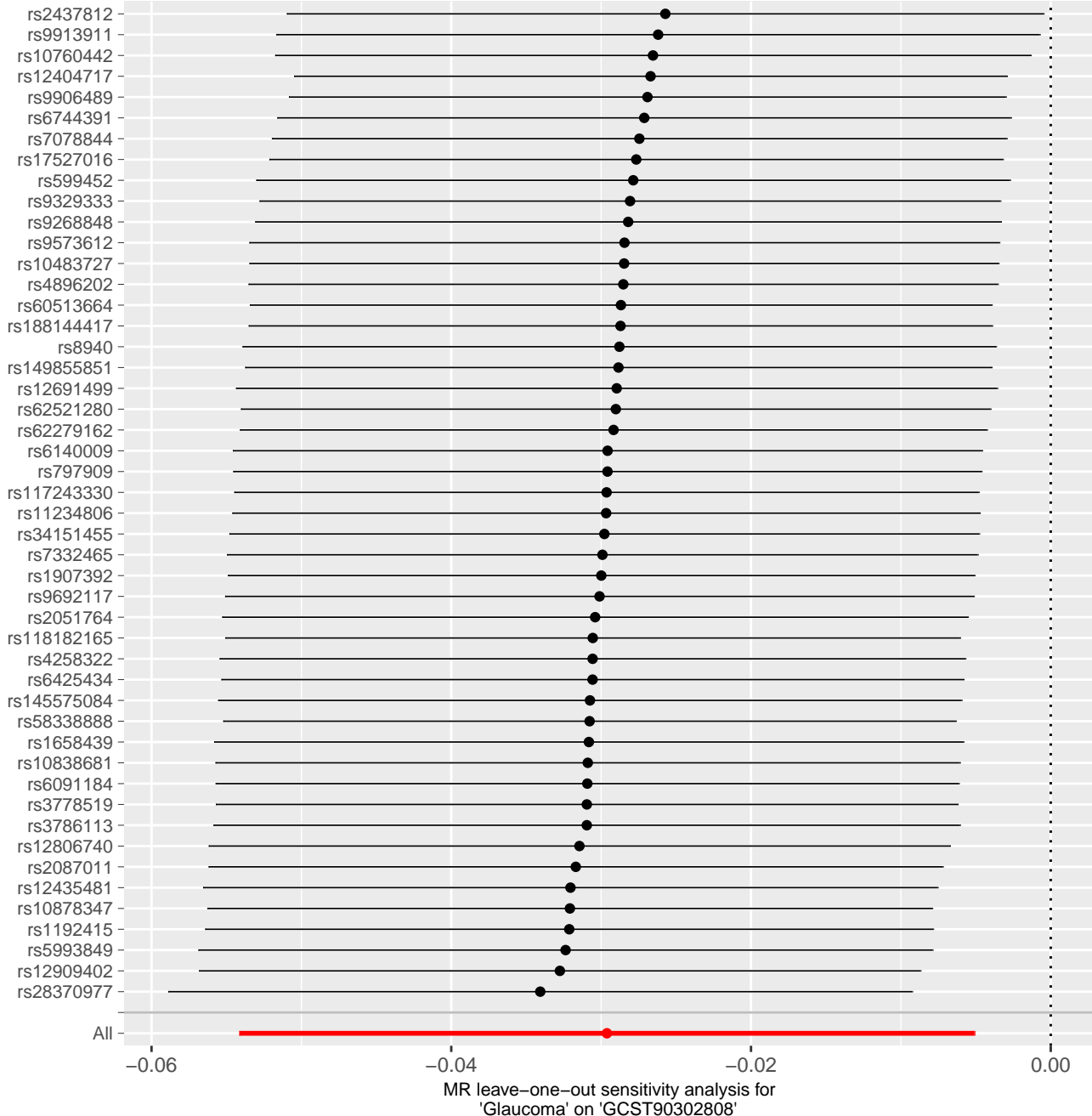

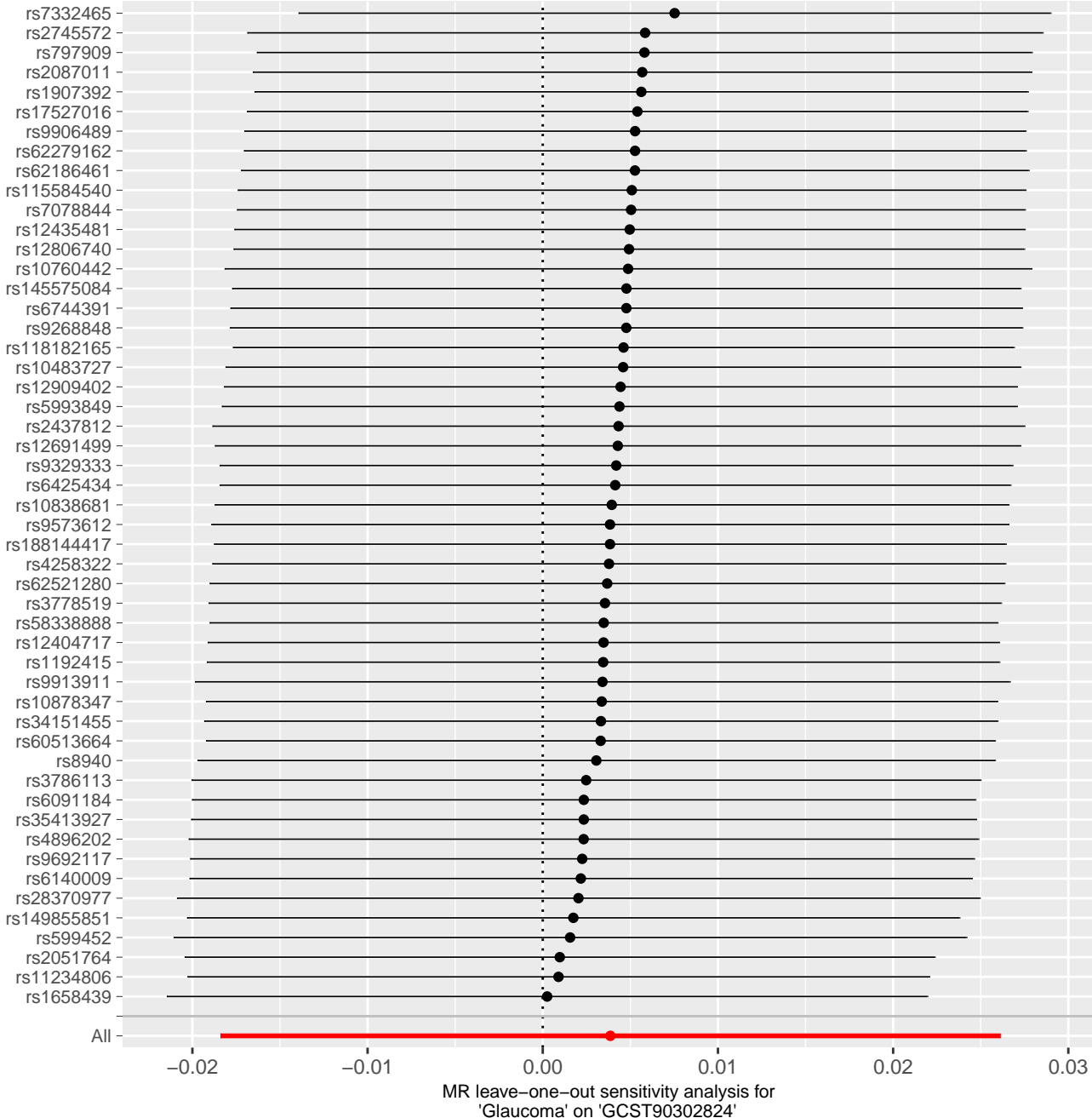

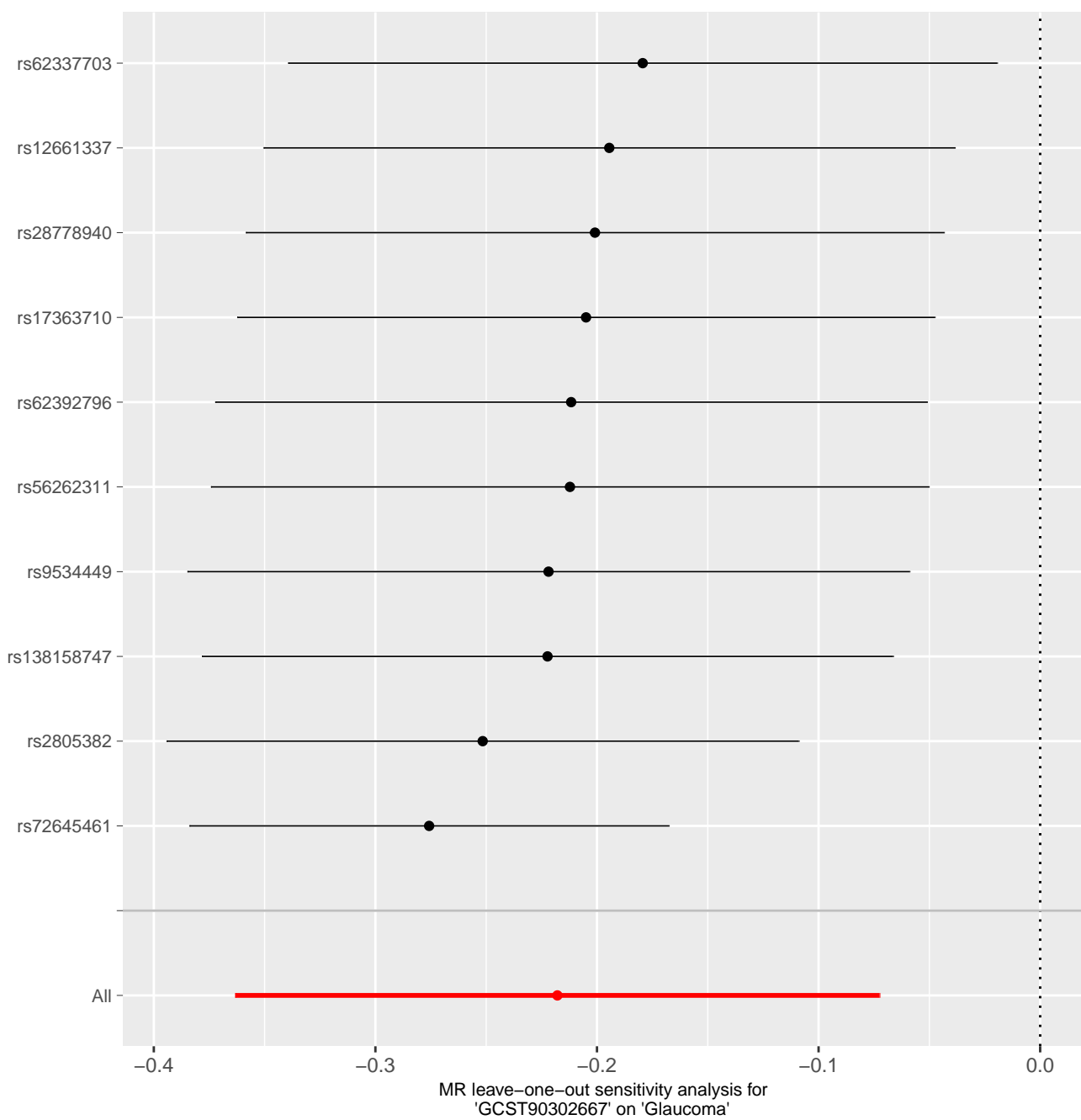

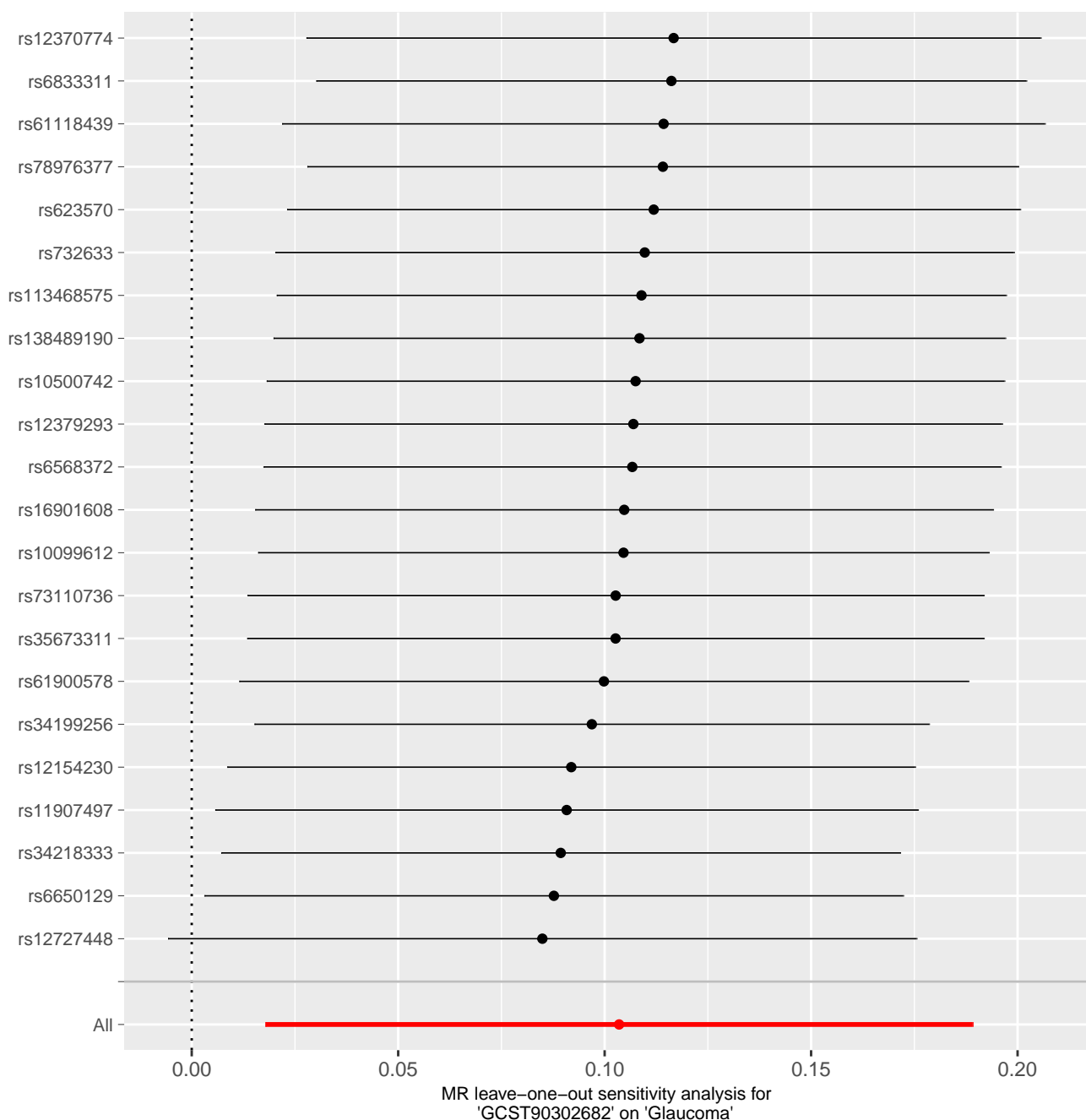

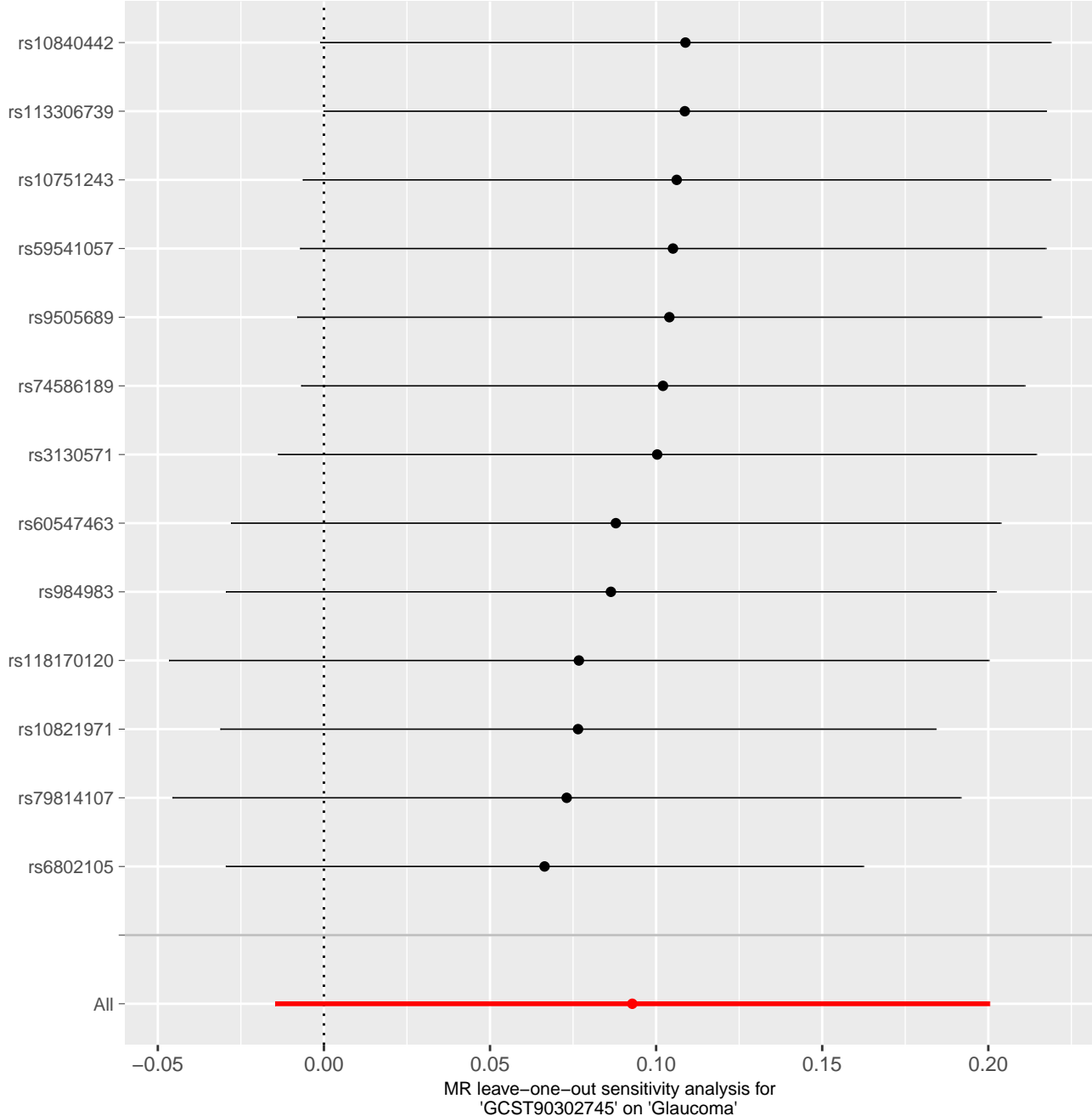

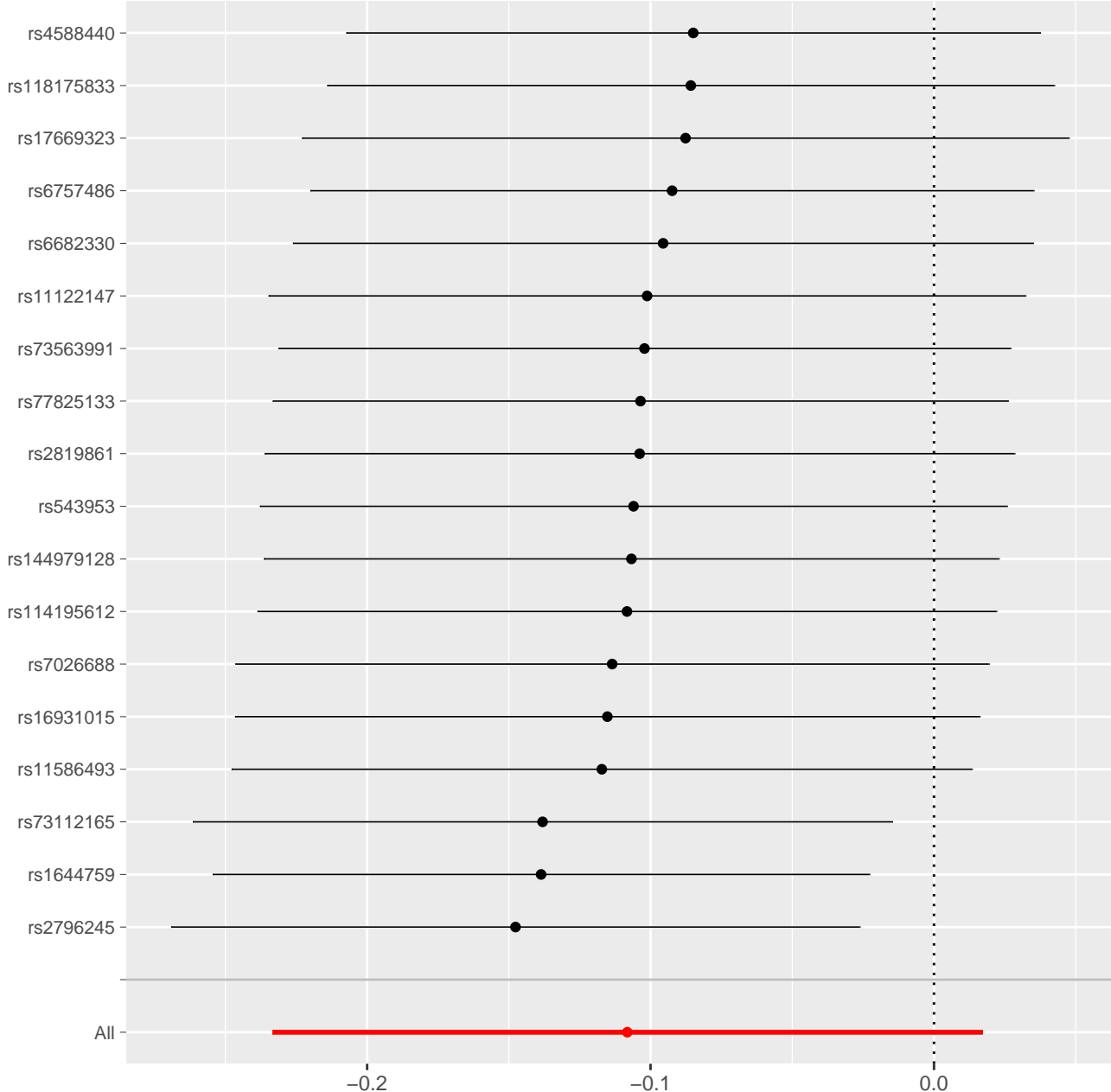

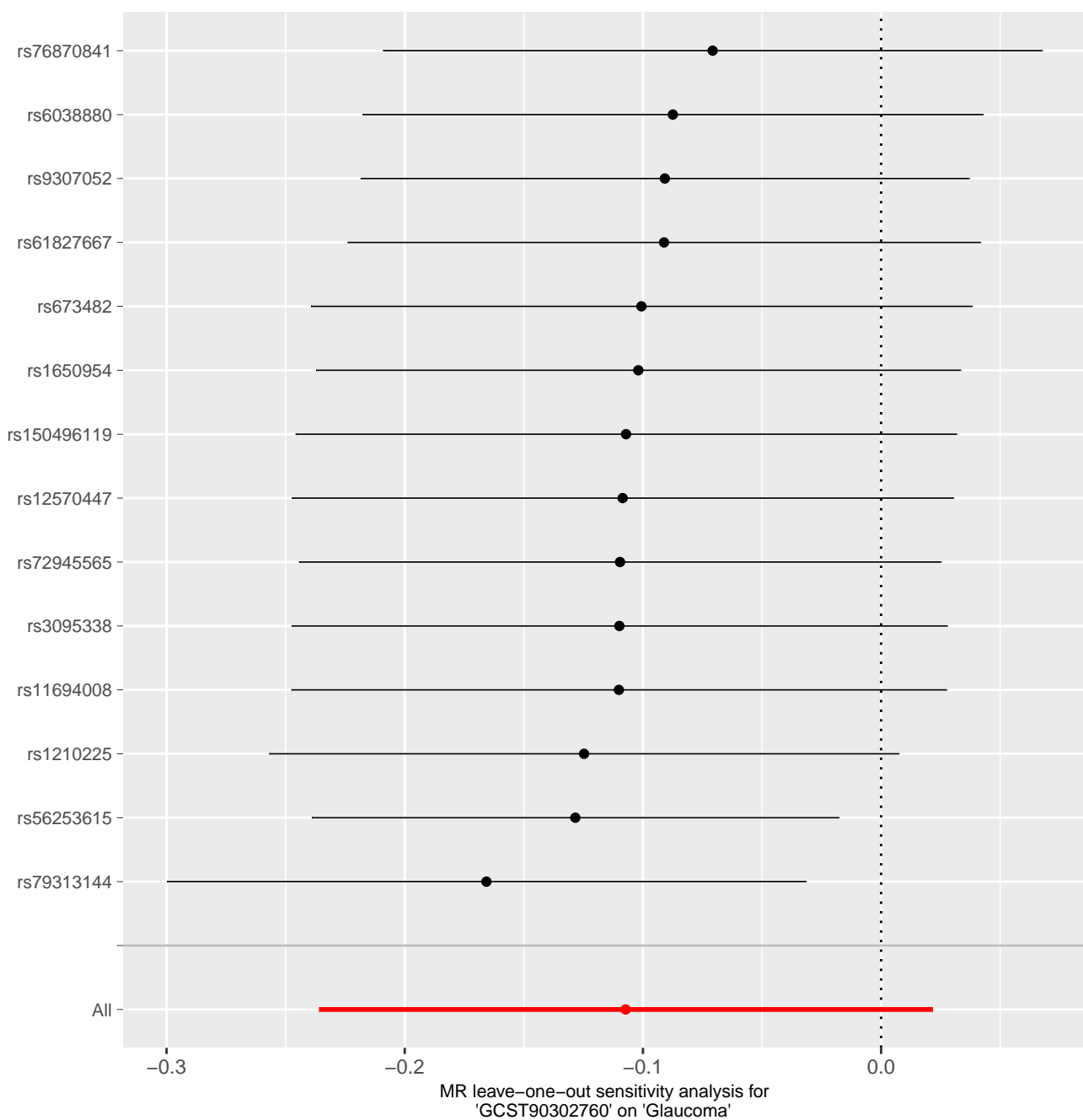

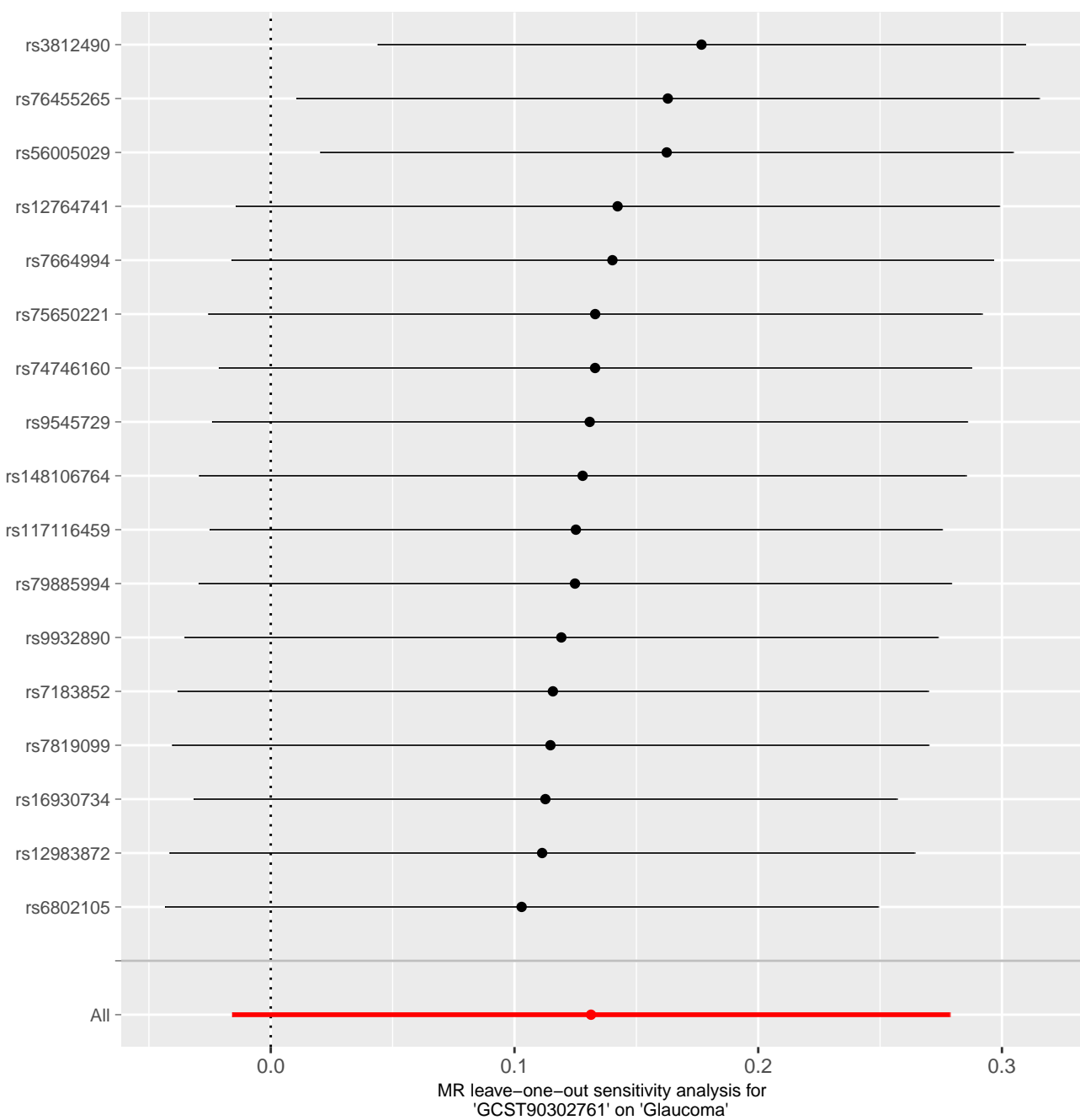

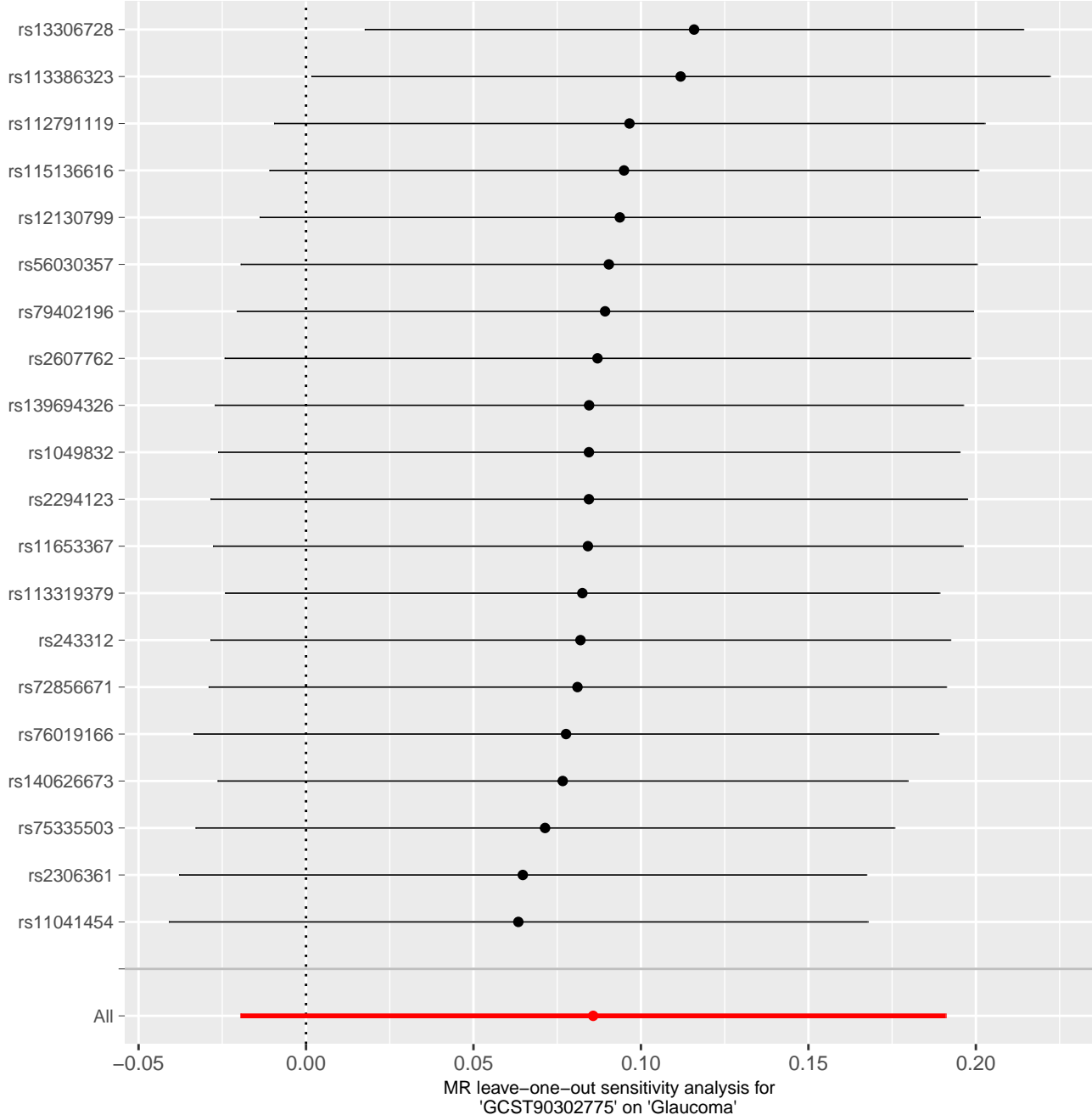

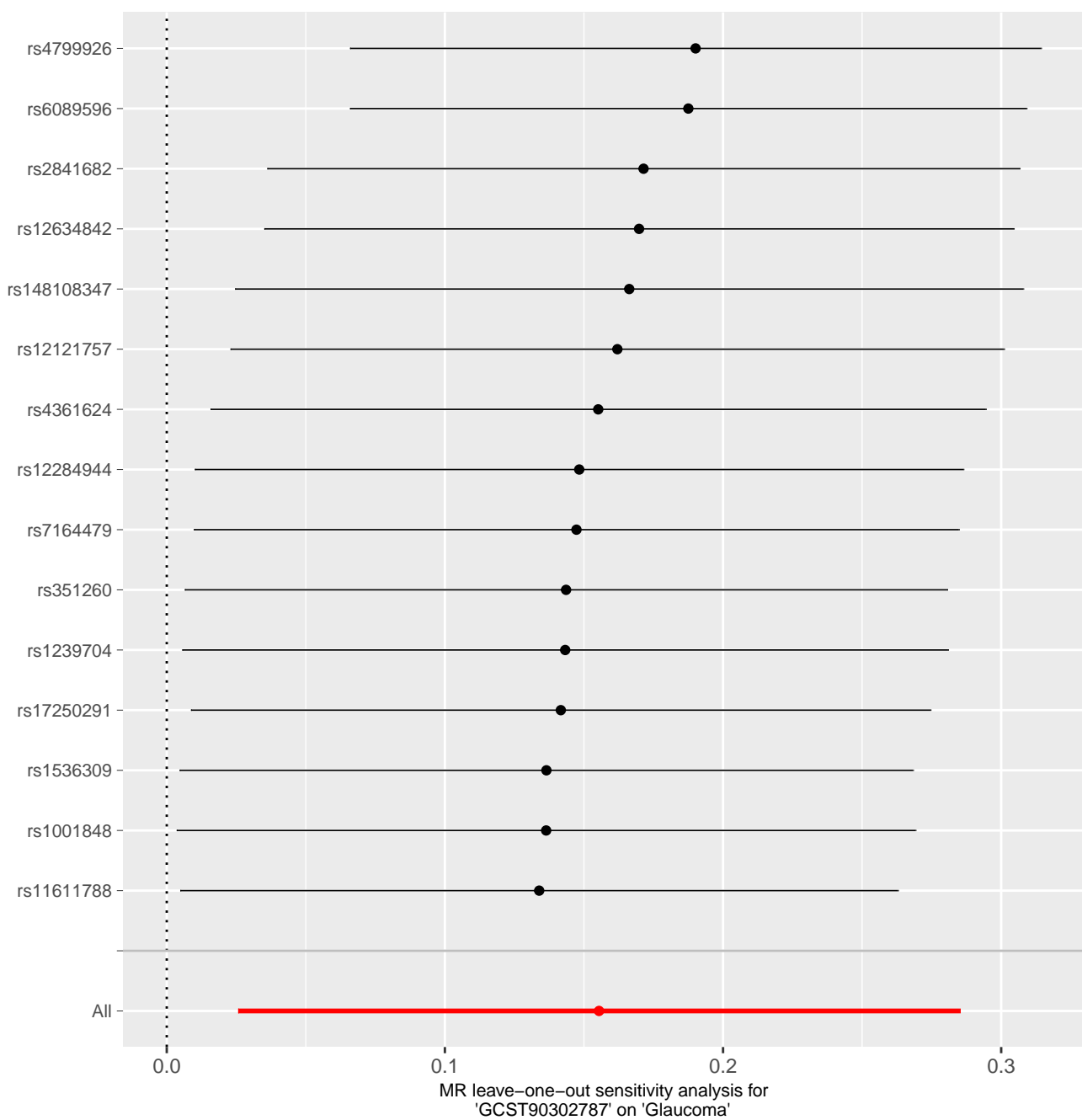

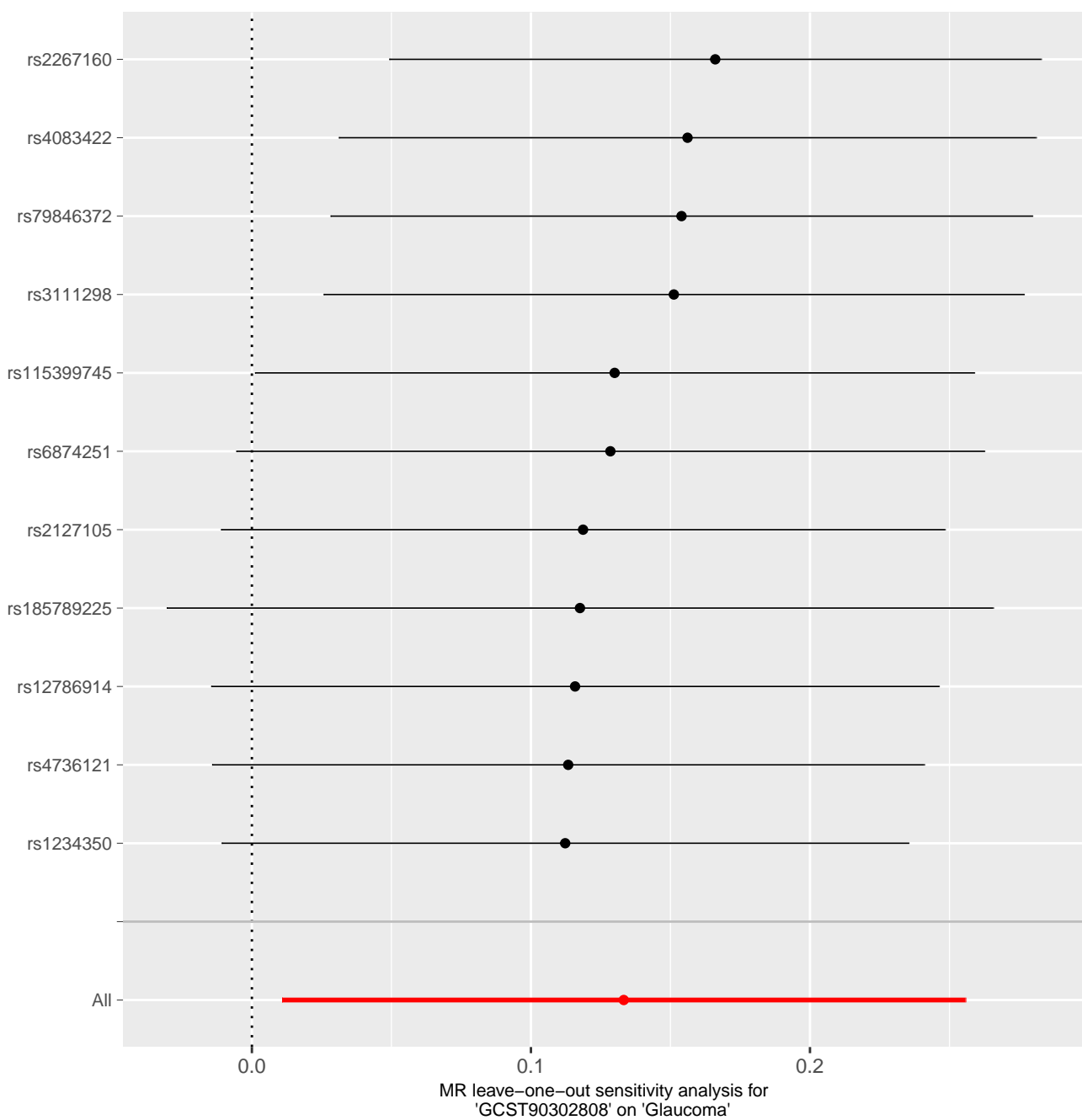

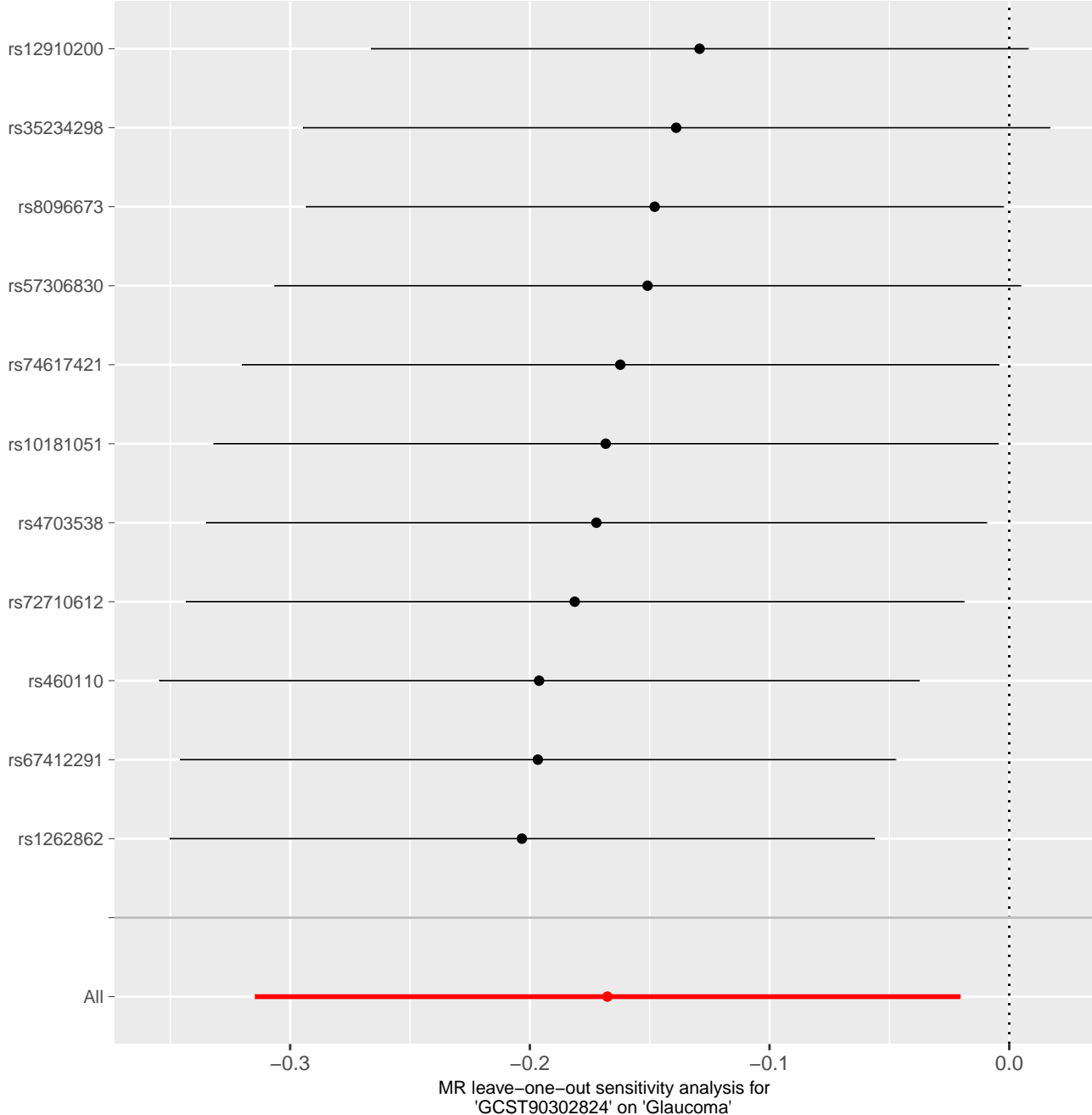

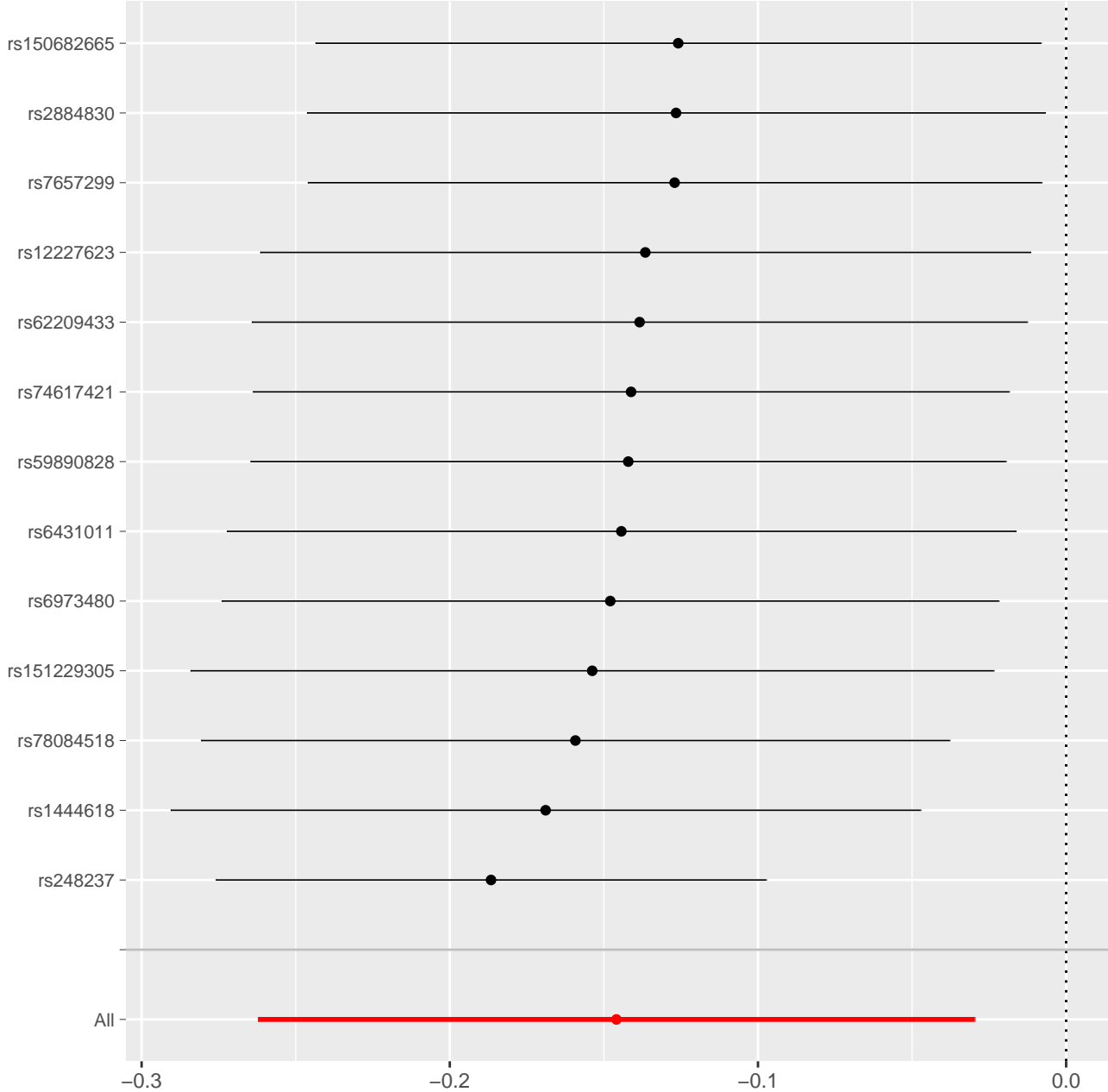

MR leave-one-out sensitivity analysis for  
'GCST90302825' on 'Glaucoma'

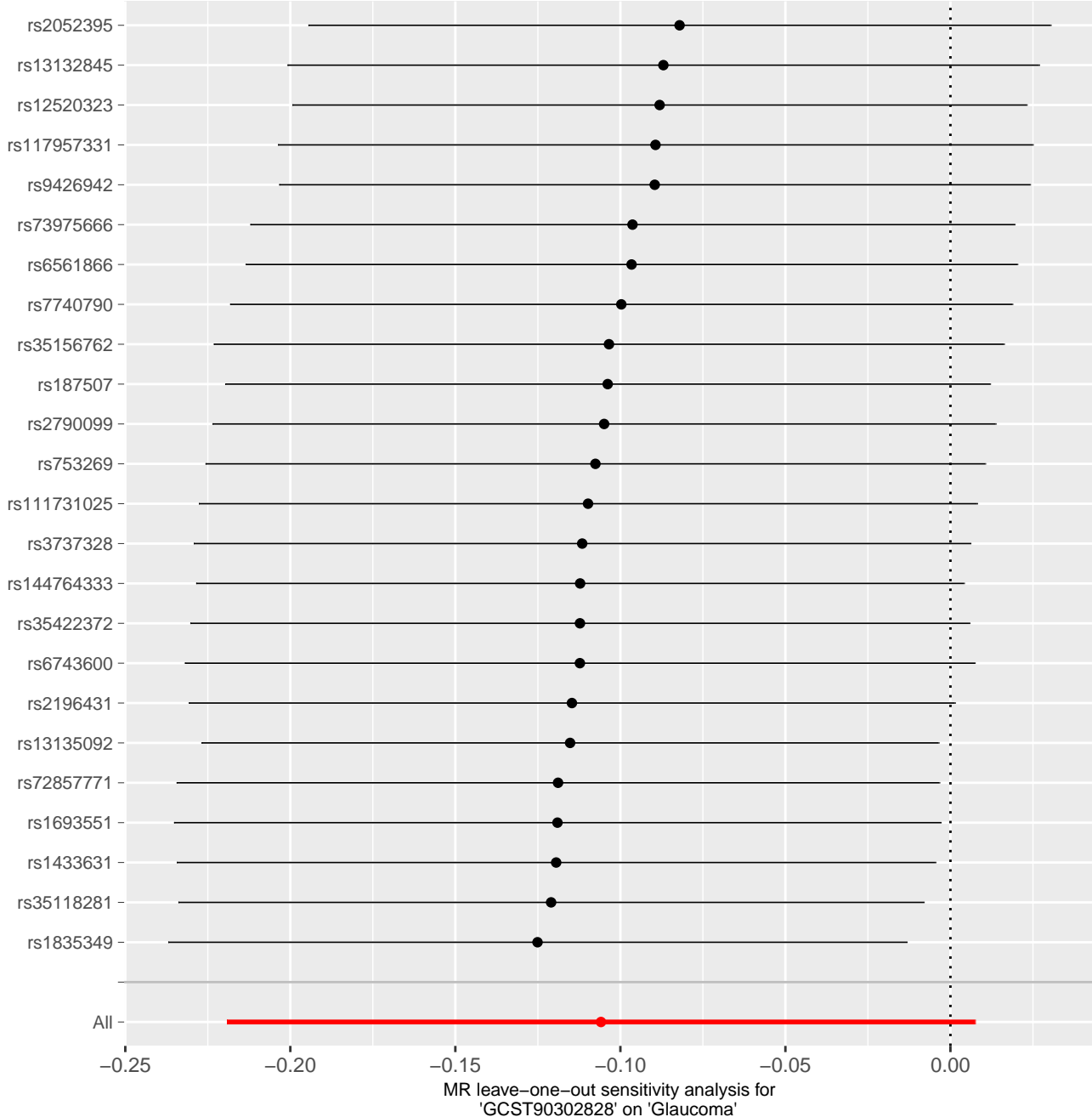

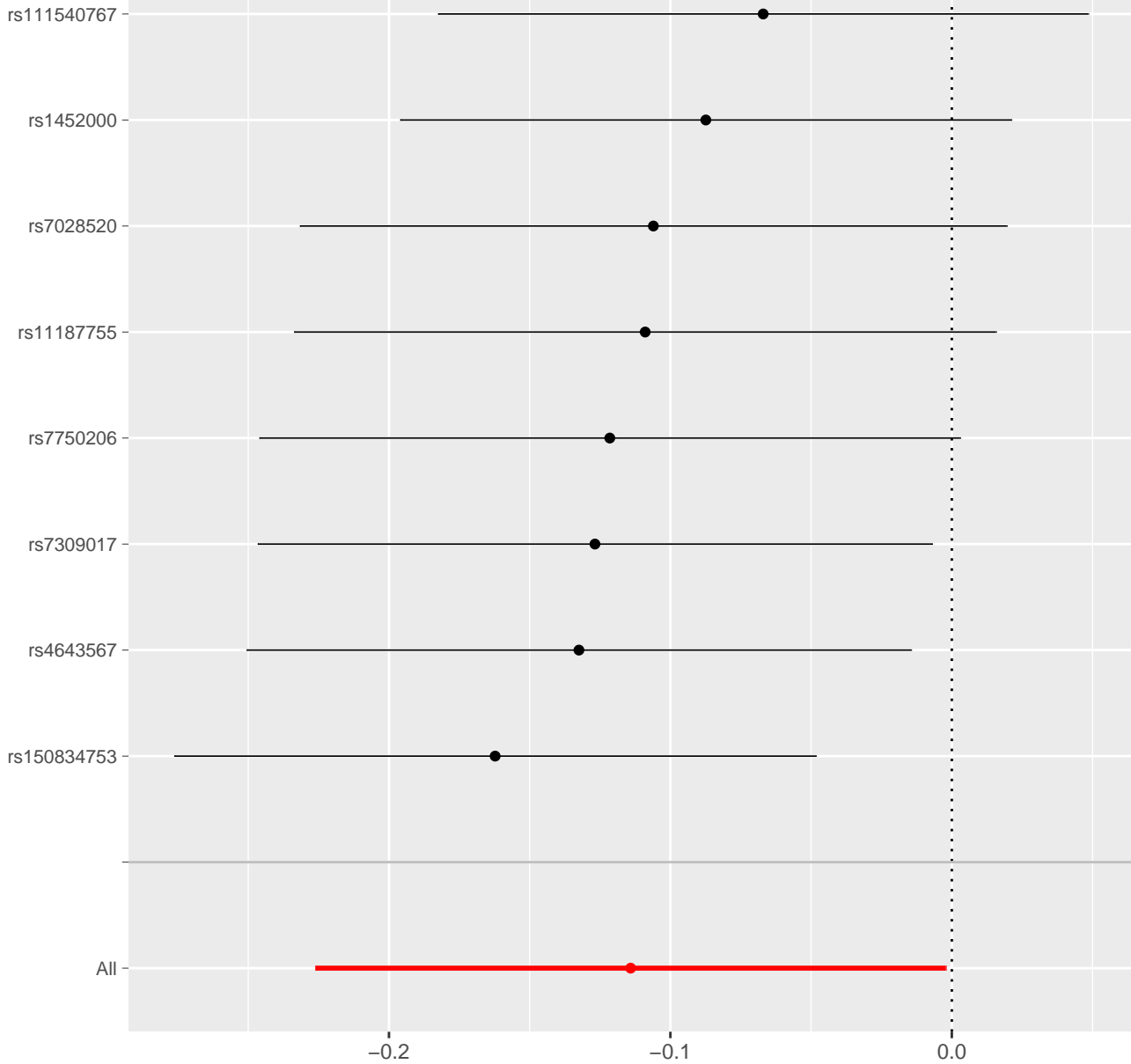

Supplement: Supplementary file 1 [file brainsci-14-01030-s001.zip › Supplementary File S1.pdf]
